# Housekeeping while brain's storming Validation of normalizing factors for gene expression studies in a murine model of traumatic brain injury

**DOI:** 10.1186/1471-2199-9-62

**Published:** 2008-07-08

**Authors:** Hervé Rhinn, Catherine Marchand-Leroux, Nicole Croci, Michel Plotkine, Daniel Scherman, Virginie Escriou

**Affiliations:** 1 Inserm, U640, Paris, F-75006 France; CNRS, UMR8151, Paris, F75006 France; Université Paris Descartes, Faculté des Sciences Pharmaceutiques et Biologiques, Chemical and Genetic Pharmacology Laboratory, Paris, F-75006 France; Ecole Nationale Supérieure de Chimie de Paris, Paris, F-75005 France; 2 Unité de pharmacologie de la circulation cérébrale, Université Paris Descartes, Faculté des Sciences Pharmaceutiques et Biologiques, Paris, F-75006 France

## Abstract

**Background:**

Traumatic brain injury models are widely studied, especially through gene expression, either to further understand implied biological mechanisms or to assess the efficiency of potential therapies. A large number of biological pathways are affected in brain trauma models, whose elucidation might greatly benefit from transcriptomic studies. However the suitability of reference genes needed for quantitative RT-PCR experiments is missing for these models.

**Results:**

We have compared five potential reference genes as well as total cDNA level monitored using Oligreen reagent in order to determine the best normalizing factors for quantitative RT-PCR expression studies in the early phase (0–48 h post-trauma (PT)) of a murine model of diffuse brain injury. The levels of 18S rRNA, and of transcripts of *β*-actin, glyceraldehyde-3P-dehydrogenase (GAPDH), *β*-microtubulin and S100*β *were determined in the injured brain region of traumatized mice sacrificed at 30 min, 3 h, 6 h, 12 h, 24 h and 48 h post-trauma.

The stability of the reference genes candidates and of total cDNA was evaluated by three different methods, leading to the following rankings as normalization factors, from the most suitable to the less: by using *geNorm *VBA applet, we obtained the following sequence: cDNA(Oligreen); GAPDH > 18S rRNA > S100*β *> *β*-microtubulin > *β*-actin; by using NormFinder Excel Spreadsheet, we obtained the following sequence: GAPDH > cDNA(Oligreen) > S100*β *> 18S rRNA > *β*-actin > *β*-microtubulin; by using a Confidence-Interval calculation, we obtained the following sequence: cDNA(Oligreen) > 18S rRNA; GAPDH > S100*β *> *β*-microtubulin > *β*-actin.

**Conclusion:**

This work suggests that Oligreen cDNA measurements, 18S rRNA and GAPDH or a combination of them may be used to efficiently normalize qRT-PCR gene expression in mouse brain trauma injury, and that *β*-actin and *β*-microtubulin should be avoided.

The potential of total cDNA as measured by Oligreen as a first-intention normalizing factor with a broad field of applications is highlighted. Pros and cons of the three methods of normalization factors selection are discussed. A generic time- and cost-effective procedure for normalization factor validation is proposed.

## Background

Real-time RT-PCR, which allows to measure any chosen RNA with great accuracy over a large dynamic range, has become the gold-standard for nucleic acid quantification. It has also opened new investigations fields, since very small amount of RNA is needed, allowing transcripts from low-expressed genes or from very small samples to be quantified.

If constant developments in both reagents and data analysis make real-time PCR measurements more and more accurate and reliable, many considerations have to be taken to convert this technical precision into biologically relevant data. Factually, real-time RT-PCR gives access to the number of copies of a chosen sequence in a cDNA solution, which is obtained from RNA extracted from a known quantity of tissue. The biologically relevant information that has to be ultimately obtained is the global expression level of the chosen gene in the tested sample, at least relatively to another sample.

The quantification of a target gene in a given sample needs three majors steps: RNA/mRNA extraction, reverse transcription of the extracted RNA, and qPCR (quantitative PCR) processing of the synthesised cDNA. A control normalization may be performed at each step to level out dissimilarities between samples [1].

The first possible normalization is by equalizing samples size, such as cell number or tissue weight. This is the easiest and the most intuitive measure. However, its position upstream of the reactions sequence does not allow to correct for the distortions generated by downstream manipulations, especially by RNA extraction, whose efficiency may broadly vary from one sample to another.

The second method consists in normalizing samples according to RNA content after its extraction. This however does not take into account the reverse transcription efficiency, known to vary from one sample to the other [2].

Thus, a downstream normalization method appears to be the most effective. This may be performed by measuring the expression level of a gene transcript expressed in the sample, as an endogenous control for the different reaction steps. The housekeeping term, which is often applied to these genes, was initially given to genes that are necessary for the function of each cell. As a matter of fact, they have to be expressed in each cell type. The most typical case is *β*-actin, a cornerstone of the inner architecture of the cell. This makes housekeeping genes suitable for organism-wide positive controls for many cDNA-based techniques, but does not ensure their expression levels to be equals. The expression levels of usual housekeeping genes has however been shown to vary in some conditions [1].

If the belief in the existence of perfect reference genes, whose levels would remain unchanged in each cell whatever the tissue or the experimental conditions, is known to be more idealistic than real, reference genes have to be chosen specifically for a given experiment, on the basis of the stability of their expression in the subset of studied tissues and experimental conditions one is interested in. In consequence, the use of internal controls implies a proper validation for each experimental condition, as the use of unappropriate normalizing factors, with unconstant expression levels, would generate discrepancies in normalized expression results [3]. Several methods have been described for that purpose [4-7].

In addition to the use of reference genes, alternative normalization procedures have been proposed for RT-PCR: the addition of known quantity of artificial RNA molecules to extracted RNA prior to RT reaction [8], or the use of an oligo-dT linked artificial target sequence quantifiable by quantitative PCR (qPCR) [9] or the total cDNA quantification by fluorescent dyes [10].

In the specific context of brain trauma, sample size measurement is especially doubtful, since the oedema resulting from the trauma [11] may enhance brain water content, hence lowering the cellular density of a given weight of tissue. This effect may also be strengthened, as necrosis and apoptosis are known to be major post-trauma events [12,13].

We have reviewed the use of PCR to quantify mRNA levels in mouse and rat traumatic mechanical brain injury models for the five last years. We have identified 22 qPCR and 17 semi quantitative PCR studies. About 85% of them used a reference gene for normalization, while the others only used RNA quantification for this purpose.

GAPDH has been up to now the most commonly used reference gene in qPCR [14-24] and the second one [25-28] for sqPCR (semi-quantitative PCR). The use of *β*-actin has been predominant for sqPCR [29-36], but less frequent for qPCR, to the benefit of 18S rRNA [37-41]. We have found no correlation between the choice of a reference gene and the pathological model (Controlled Cortical Injury (CCI), Closed Head Injury (CHI) or Fluid Percussion Injury (FPI)) or species. Strikingly, a single reference gene has been used in all the experiments, without reference to a prior validation, whereas the use of more than one reference gene is widely encouraged [4], and the need for validation highlighted [1,3]. Indeed, the choice of reference genes is especially crucial for brain trauma studies, where many biological pathways are implicated (inflammation, hypoxia, apoptosis, neovascularisation...), resulting in numerous gene expression changes, which are thus likely to include or affect potential reference genes.

The present study aims at the validation of optimal nomalizing factors(NF) for RT-qPCR-based transcriptional studies of an early phase of a murine model of traumatic diffuse brain injury.

RT-qPCR was used to determine the expression levels of five potential reference genes. Total cDNA level was also measured using Oligreen reagent and considered as an other potential NF. These six candidates were compared using different NF-selection methods.

## Results

### Reference genes and total cDNA level

After induction of trauma, 10 mice were sacrificed at 30 min, 3 h, 6 h, 12 h, 24 h and 48 h, and RNA was extracted from the lesionnal zone of injured brains or from the equivalent zone of uninjuried control mice brains, then reverse transcribed. The expression level of 18S rRNA, *β*-microtubulin, S100*β*, *β*-actin and GAPDH were measured by real-time PCR for each individual cDNA.

**Figure 1:**
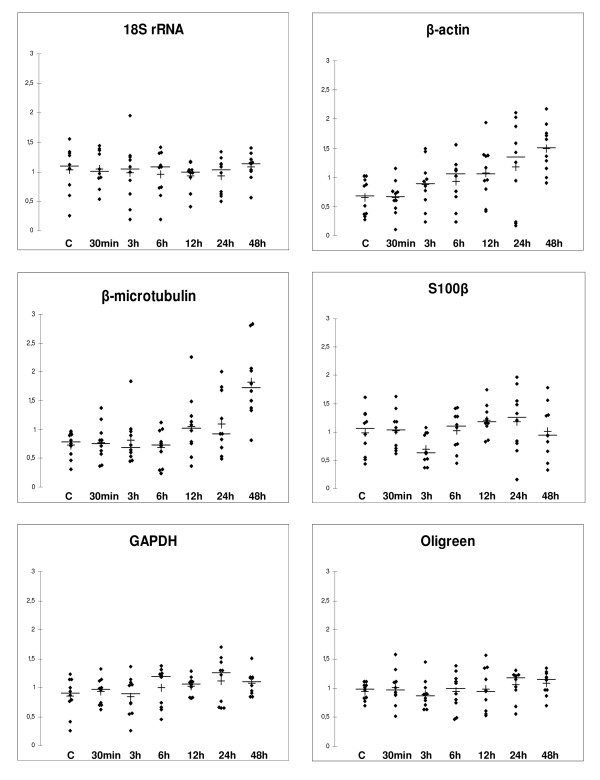
**Level of normalizing factors at various time post-trauma**. The levels of 18S rRNA, *β*-microtubulin, S100*β*, *β*-actin and GAPDH as measured by real-time PCR, and cDNA level as measured with Oligreen were determined at the indicated time point post-trauma. Each point corresponds to the measured level for one individual sample, normalized to the mean of all individual values at all times Horizontal bars represent the median of the measures for the group, vertical crosses represent the arithmetic mean. (C): control.

Oligreen reagent is a dye that emits fluorescence at 500/520 nm when bound to single-stranded DNA, but is insensitive to free nucleotides and to very short oligonucleotides. However, Oligreen emits fluorescence in the presence of RNA at room temperature; its use for cDNA quantification therefore implies to work at 80°C to specifically quantify reverse-transcribed cDNA [43]. Levels of reference genes at different time post-trauma, as measured by qPCR and of total cDNA as measured using Oligreen reagent, are represented on Fig. 1.

### ANOVA

A statistical test was applied to look for significant differences between two experimental conditions for each NF level. As the variance equality hypothesis was verified for each of them (no significant variance differences detected with a Hartley test for a confidence level of 95%), a one-factor ANOVA test was used, with a Fischer's test to detect significant differences (p < 0.05) between two groups, for a given NF expression level.

The expression level fluctuations between time groups were found to be significant for *β*-actin, S100*β *and *β*-microtubulin. No significant differences were detected from one group to another for GAPDH, 18S rRNA and cDNA (Oligreen).

Significant differences between experimental groups clearly make the tested NF unsuitable to normalize sample of the tested experimental set. The expression level of *β*-actin, S100*β *and *β*-microtubulin should thus be avoided as a NF for the present experimental model.

To further study the suitability of the studied normalization factors, three published selection methods were applied: geNorm analysis [4], Normfinder analysis [6] and a confidence interval based method [7].

### GeNorm analysis

**Figure 2:**
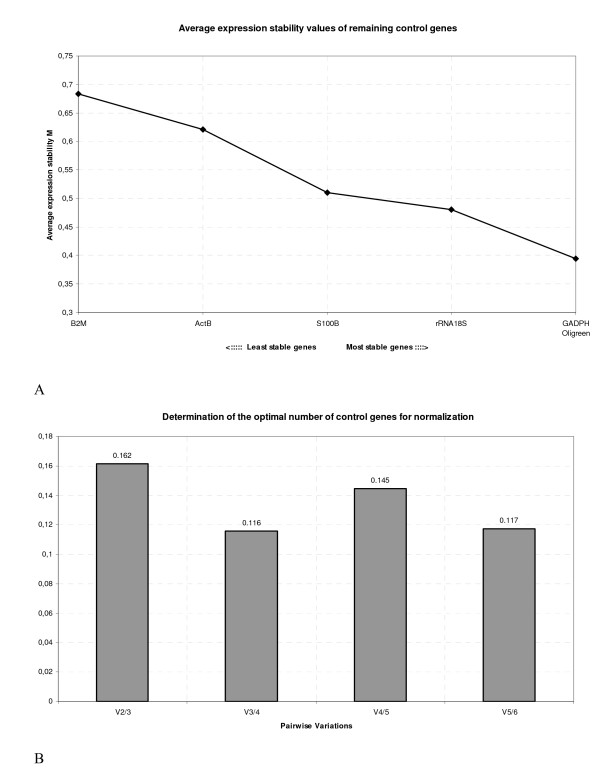
**Genorm output**. **A: **Expression stability values (M) of the candidate normalization factors, during the stepwise exclusion of least stable normalization factors. Candidate normalization factors are ranked from the least stable to the most stable (left to right). **B: **Determination of the optimal number of normalization factors to be used, based on the analysis of pairwise variation (V) between normalization factors. A low value of V means that the inclusion of an additional normalization factor would not lead to a increase in stability.

Gene stability analysis was performed using the geNorm VBA applet as described in [4]. Briefly, the basic assumption of this method is that the ratio of two perfect reference genes should be constant throughout the different experimental conditions. The inter-condition variability of this ratio is thus evaluated for each experimental condition and for each couple of reference gene, and a gene-stability-measure M is calculated for each candidate. This reflects the average pair-wise variability between one putative reference gene and all the others. The less stable candidate (i.e. with the highest M value) is then excluded as the least suitable NF, and a new step of M-values calculation has then to be performed because the former inclusion of the less stable gene had influenced other M values. By sequential exclusions, the two most-stably expressed genes are selected. The ranking of the studied NF according to their M value, as calculated by the GeNorm software was, from the most stable to the least:

GAPDH – cDNA(Oligreen)>18S rRNA > S100*β *> *β*-actin > *β*-microtubulin (Fig. 2A).

As proposed by Vandersompele et al. [4], the use of a geometric mean of multiple NF among the best ranked should ensure a more accurate normalization. The choice of NF to be included starts from the most stable NF, by a step wise inclusion of the next-ranking NF and the influence of the inclusion on the overall stability is plotted by the GeNorm software (Fig. 2B). A strong instability elevation implies that the inclusion is detrimental, and that the last included NF should thus be discarded. In our case, GeNorm method leads to the use of the geometric mean of total cDNA and GAPDH levels for the best normalization.

#### - Normfinder analysis

A model-based approach, was used as an another technique to rank the best potential reference genes, using the Excel Spreadsheet Normfinder [6]. The criteria for sample sizes (n>8) and candidate RG (n>3) were met. Briefly, for one given sample and one given candidate reference gene, the log-transformed measured level is formulated as the sum of three terms: the general expression level of the gene in the experimental group to which the sample belongs, the amount of mRNA in the sample, and the random variation caused by biological and experimental factors. The latter term is used to calculate an intra-group variation, while the first term permits to evaluate an inter-group variability for the considered NF. Both variations estimations are combined in a stability value used to rank the NF.

**Figure 3:**
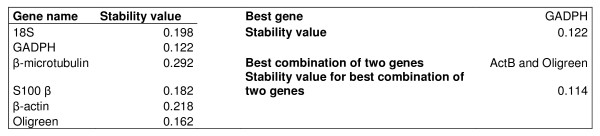
Normfinder stability values output.

The ranking of each NF according to its stability value as calculated by the Normfinder software was, from the most stable to the least:

GAPDH>cDNA(Oligreen)> S100*β *>18S rRNA > *β*-actin > *β*-microtubulin (Fig. 3).

#### - Confidence-Interval method

A third approach has been used, based on the one presented in Haller et al. [7]. Equivalence testing is similar to classical statistical tests, but while most of them are based on the rejection of a relative closeness hypothesis to prove a significant difference between two means, equivalence testing relies on the rejection of an hypothese of a relative difference between two means to prove their significant closeness.

This can be tested by classical t-test, but, for a given level of confidence alpha, one can proceed more visually through a confidence interval calculation, which is the most-used method for statistical assessment of clinical trials.

Briefly, two samples T_i _and T_j_, with expression levels *μ*_i _and *μ*_j_, are considered to be equivalent for a confidence level alpha if their confidence interval I_ij_, centered on *μ*_i _- *μ*_j _and whose length is based on a Student t-distribution depending of the distributions of the two samples (detailed in Haller et al.) is such as *I*_*ij *_⊂ [-*ε*, *ε*], epsilon being an arbitrarly chosen treshold. Moreover, if 0 ∉ *I*_*ij*_, the two groups are significantly different for the confidence level.

When dealing with expression levels, intrinsic meaning has to be found in ratio rather than in absolute difference. We thus worked with log-transformed expression levels for equivalence testing. In this way, the limit epsilon that has to be chosen is equivalent to a fold-change. We chose to set it to 1, which means an expression ratio between the two compared groups inferior to two.

**Figure 4:**
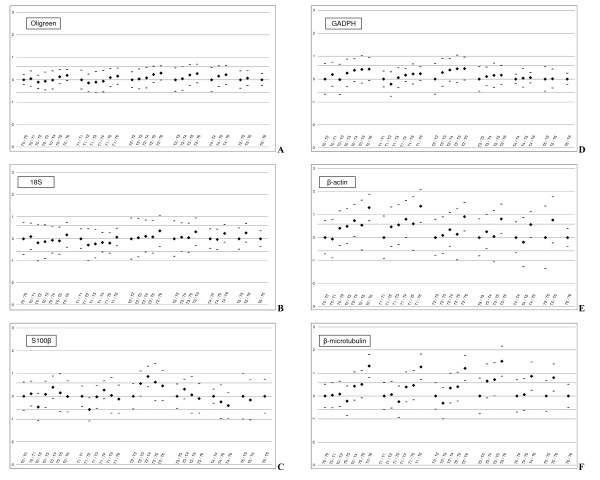
**95% Confidence intervals for equivalence between experimental groups**. T_G_/T_G' _corresponds to the interval calculated between group G and group G'. With T0: control group and T1, T2, T3, T4, T5 and T6 trauma groups sacrificed at 30 min, 3 h, 6 h, 12 h, 24 h and 48 h respectively. For symmetrical reasons, since I_ij _= I_ji_, only (*I*_i,j_)_i≤j _are plotted. For each of the normalization factor: If 0 ∉ *I*_*ij*_, its level is significantly different between experimental conditions i and j. If *I*_*ij *_⊂ [-*ε*; *ε*], its level is equivalent between experimental conditions i and j. **A: **Oligreen measurements; **B: **18S rRNA; **C: **S100*β*; **D:**GAPDH; **E: ***β*-actin; **F: ***β*-microtubulin.

The confidence intervals for log-transformed normalized NF levels were calculated for each couple of experimental groups and for all NF, with 95% confidence levels (Fig. 4). Two groups were said to be equivalent with 95% confidence if their confidence level is included in [-1;1].

Measured levels were found to be equivalent between all groups for cDNA(Oligreen).

**Figure 5:**
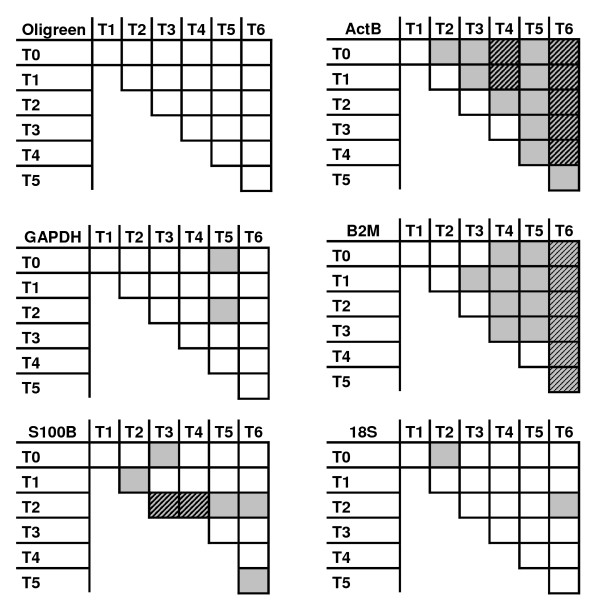
**Schematic results for confidence interval calculations**. Confidence interval are calcultated using a cut-off fold change = 2 and 95% confidence level. White squares: the two groups are equivalent for the considered NF. Grey squares: the two groups are not equivalent for the considered NF. Dashed squares: the two groups are significantly different for the considered NF. With T0: control group and T1, T2, T3, T4, T5 and T6 trauma groups sacrificed at 30 min, 3 h, 6 h, 12 h, 24 h and 48 h respectively.

For 18S rRNA and GAPDH, the expression levels were found not to be equivalent for two couples of groups out of twenty-one. It was the case for 8 combinations for S100*β*, 18 for *β*-microtubulin and 19 for *β*-actin (Fig. 5) An equivalence-based ranking thus appears to be:

cDNA(Oligreen)> 18S rRNA-GAPDH> S100*β *> *β*-microtubulin > *β*-actin

## Discussion

We have used the Oligreen reagent to quantify the cDNA effectively synthesised during the RT step. Whatever the procedure for NF choice, cDNA as measured by Oligreen ranked among the two best. As its use appears to be validated for the present model, it should also be very attractive for other cases, since small variability is unlikely to depend on experimental conditions, in contrast to other reference genes.

**Figure 6:**
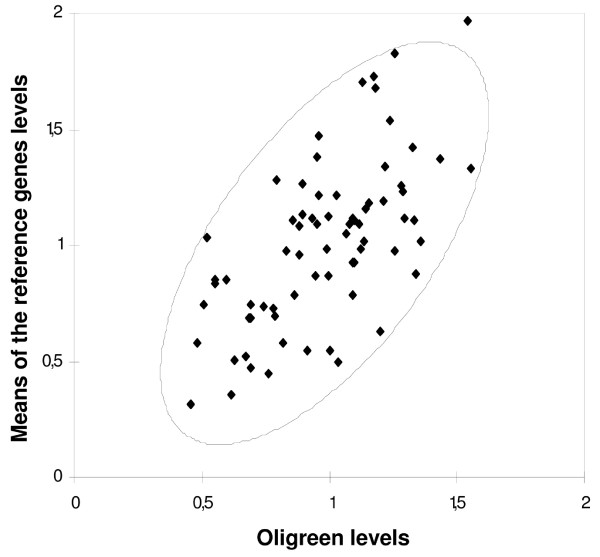
**Correlation between Oligreen measurements and other reference genes**. Each point corresponds to one sample, as the mean of normalized measured levels for all tested reference genes in function of the normalized measured levels of Oligreen. A 95% confidence ellipse is drawn.

To ensure that the overall high stability of Oligreen-assayed cDNA is not an artefact, we compared for each sample the mean of all studied reference genes levels to the measured Oligreen level. The aim is to ensure that Oligreen reflects the level of cDNA in each sample. The application of the previously used selection procedures for RNA input – equal by definition to 1 *μ*g for each sample – would indeed have led to the systemic selection of RNA quantification as the best normalizing factor, with both intra- and inter- group variation equal to zero. RNA level is however obviously not an appropriate normalizer, as it does not encompasses RT efficiency. As the mean of all the gene expression levels measured is likely to be proportionnal to the cDNA content, studying the correlation between this average expression level and the Oligreen level for each sample appears as a good mean to evaluate whether Oligreen measurement is proportionnal to the cDNA content or not. A significant correlation was found using a Pearson test (p-value < 0.0001, Pearson coefficient: 0.64) and the correlation plot (Fig. 6) shows that Oligreen measurements effectively reflect cDNA levels in the samples, as it correlates well with the the mean of different genes expression levels.

Oligreen thus appears to be a good candidate for a generic NF in quantitative RT-PCR experiments. Its use may be furthermore refined with alternative RT protocols, such as an oligo dT-primed cDNA synthesis. In such a case, Oligreen would reflect the level of effectively reverse-transcribed mRNA, in contrast to total RNA in the present study.

The use of Oligreen combined to a standard curve of known DNA concentration could moreover allows a precise cDNA quantification. In parallel to an absolute quantification of target genes, one could thus formulate the expression level of a studied gene, as the number of copies per microgram of cDNA, which would have a stronger intrinsic meaning and could open the gate for easier intra-experiments meta-analysis.

The question remains open whether the fact of combining Oligreen measurements to the expression level of internal control (18S rRNA and GAPDH in our case) brings more accuracy.

Following a confidence-interval based methodology, Oligreen appeared as the only NF for which all groups were found to be equivalent to each other, but one has to keep in mind the arbitrary choice of fold-change cut-off level and confidence levels: 18S rRNA and GAPDH appear to be very close to equivalence and thus appear as acceptable normalizing factors.

When working with confidence levels, an arbitrary level of fold change has to be set. A possible criterion for assessing the pertinence of a chosen cut-off fold-change may come from the observation of confidence intervals I_ii _calculated for a group with itself: as a group is trivially equivalent to itself, the calculated confidence interval is nothing but the reflection of the group's standard deviation. It would thus make little sense to choose a cut-off fold change that would not lead to the conclusion that a group is equivalent to itself. This consideration excluded the initially-considered choice of 1.5 as a cut-off fold-change in our case, and led to the use of 2 as a cut-off fold-change for the study. A standardized confidence interval approach, which remains to be developped, should thus set the cut-off fold change in function of the intra-groups confidence intervals. One has however to keep in mind that alpha level is arbitrary too, and that its value has a direct effect (through t-distribution) on the interval size: lowering cut-off fold change is in some extent equivalent to setting a higher confidence level alpha. These two values have thus to be chosen in a concerted manner to make sense.

Among the different procedures of normalizing factors that were applied, two criteria appear to be determinant. On one hand, one criterion is whether the method takes into account the belonging of a sample to an experimental group (either a tissue, a differentially treated sample, or a given point of a time series). This should ultimately be a requirement, as the final goal of many, if not all, gene expression experiments consists in levels comparison between different groups. This is handled by model-based and confidence intervals based methodology, but not by pair-wise approach. However, if the Normfinder software evaluates inter-group variability, its effect is combined to intra-groups variability to calculate a candidate's stability. This implies that a NF with a low variability in each group, but with an expression level clearly different in one single group, will be considered as the optimal NF, when compared to candidate with greater variability in each group but with almost no differences between groups (data not shown). This is also the case for the GeNorm software, and is probably the worst type of error that may occur while selecting a NF, as it will generate systematic errors in normalized gene expression results.

On the other hand, a second determinant criterion for eliciting a NF is the absolute or relative character of its evaluation as a potential NF. This means whether its sole expression values, as measured for every sample, are sufficient or if it has to be compared to other potential normalizing factor. The relative comparison is clearly the basis of the pair-wise method, but is also implicitly present in the model-base approach for the calculation of both intra- and inter- group variations (through the use of $\hat{\gamma }$, variance of *α*_*ig *_in the calculation of the stability value *ρ*_*i *_(Eq. C and D, [6])). The main drawback of these circular methodologies is the risk of selecting co-regulated genes. One would thus need to examinate many carefully chosen potential NF (i.e. from biological pathways as distinct as possible) in order to ensure that a majority of them is not co-regulated, the quality of the NF selection being strongly dependant of the number of studied candidates.

The equivalence interval approach enables to evaluate each NF candidate separately. This permits to imagine a sequential approach to assess reference gene: rather than gathering an as large as possible pool of potential NF candidates, then measuring their expression level and sorting them to determine the most appropriate among them, one may choose a first-intention potential NF and test it *ab nihilo*. If the test concords with previously fixed criteria, then the candidate is validated as a NF, otherwise, an additional candidate has to be tested. As it seems best to normalize with more than one reference gene, one may stop the selection procedure when two or three candidates are positively evaluated.

An additional interesting feature of an absolute evaluation is that it enables researchers to proceed to a post-hoc validation of their previously chosen NF on the sole basis of already-existing data analysis, with no need to run additional experiments with additional NF. This appears to be especially interesting when the biological material was totally used for previous reactions.

Despite its less restricted use, probably due to the lack of an easy-to-handle Excel spreadsheet as it is the case with GeNorm and Normfinder, the confidence interval based method appears to be the most powerful and most adequate for NF selection. A scoring procedure based on this approach, allowing a ranking of different NF, along with an automated easy-to-handle automated application, may be an interesting tool to develop.

## Conclusion

In conclusion, we present here the first study aiming at the identification of optimal normalizing factors in a model of traumatic brain injury. If the use of *β*-actin and *β*-microtubulin appears to be avoided, the combination of different methods leads us to suggest the use of a geometric mean of 18S rRNA, GAPDH and total cDNA as measured with Oligreen. The use of cDNA measurement with Oligreen has been validated in the present case and is encouraged as first-intention generic normalization factor. The present study also highlights the interest of using confidence interval for normalizing factor accuracy assessing, and opens the way for an iterative, time and cost-effective normalizing factor selection procedure.

## Methods

### Closed head injury model

Male CD-1 mice (IFFA Credo, France), weighting 21–24 g, were housed at room temperature under controlled light conditions (12 h light: 12 h dark) with food and water ad libitum. The murine model of diffuse head injury was used as previously described [11]. Mice were briefly anesthetized under 2% halothane balanced with air and oxygen. Closed head trauma was induced by a 50 g weight dropped from 32-cm height along a stainless steel string. This model typically results in a mortality of 30% within the first 10 min following the impact, with no observed delayed mortality. Animal care was in compliance with French regulations on protection of animals used for experimental research and with the EC regulations (Official Journal of European Community L35812/18/1986).

### cDNA preparation

Mice were sacrificed at different times (30 min, 3 h, 6 h, 12 h, 24 h and 48 h post-trauma). Brains were immediately removed. Column-shaped samples of approximately 10 mg were taken vertically around the lesion site using a 4 mm punch and put in RNAlater solution (QIAGEN), and were kept at 4°C for further extraction of total RNA using the Rneasy kit (QIAGEN). RNA concentration was assessed using a NanoDrop ND-1000 Spectrophotometer (Nanodrop). One microgram of total RNA was reverse transcribed in a final volume of 20 *μ*l containing 4 *μ*L of 5× RT buffer, 20 units of RNasin RNase inhibitor (Promega), 10 mM DDT, 100 units of Superscript II RNase H-reverse transcriptase (Invitrogen), 3 mM random hexamers (Invitrogen). The samples were incubated at 20°C for 10 min, 42°C for 30 min and 99°C for 5 min.

### Real-time PCR

**Figure 7:**
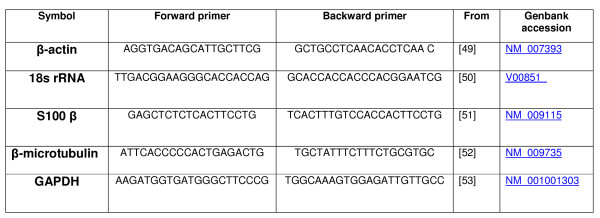
Chosen primers.

PCR primers for tested reference gene were chosen in published articles, for their common use as reference genes and their belonging to different biological pathways (Fig. 7). Real-time PCR reactions were carried out using ABI PRISM 7900HT Sequence Detection System (Applied Biosystems) in a 384-well, clear optical reaction plate with optical adhesive covers (Applied Biosystems). Reactions were run in a 5 *μ*l volume in duplicate, with 2 *μ*L of cDNA solution and 3 *μ*L of a homemade target-specific mix composed of 5/6 2× Power SYBR Green Master Mix (Applied Biosystems) and 1/6 of 100 mM primers solution. The PCR program was: 95°C for 10 min, followed by 45 cycles of (15 seconds at 95°C; 1 min at 60°C). Product specificity was assessed by 3% agarose gel electrophoresis followed by ethidium bromide staining.

For each PCR well, raw fluorescence data were exported and a curve-fitting method was applied to evaluate individual efficiencies [42]. As for each target, calculated efficiencies appeared to be homogeneous throughout samples, the efficiency for each target was considered as shared for all wells. This efficiency was set as the mean of the fitted individual efficiencies. Individual Ct values exported from the SDS software (Applied Biosystems) were used to calculate the relative expression level of target t in sample s: ${\text{L}}_{\text{t,s}}=eff_{t}^{-Ct(s,t)}$

As dealing with numerous normalizing factors, these relative expression levels were normalized for each target, for easier calculation and comparison.

### Oligreen measurements

Fluorescence measurements was carried out using ABI PRISM 7900HT Sequence Detection System (Applied Biosystems) in a 384-well, clear optical reaction plate with optical adhesive covers (Applied Biosystems). The reactions were run in a 5 *μ*l volume in duplicate, with 2 *μ*L of cDNA solution, 3 *μ*L of Oligreen stock solution (Invitrogen) 200× diluted in TE buffer. Fluorescence was continuously read at 80°C for 1 min, a condition which ensures exclusive cDNA measurement [43]. Measurements linearity was assessed by a cDNA standard curve. After background substraction, fluorescence values were normalized throughout all samples to be treated in the same way as PCR-based NF values.

### Data analysis

#### Statistical analysis

Statistical calculations were run with Microsoft Excel and XLSTAT 2006 add-on (Addinsoft).

#### Confidence interval calculation

For each reference gene, groups are tested two-by-two for their equivalences. For two groups (T_i_;T_j_) with the same number of elements (N), with means (${\hat{X}}_{i};{\hat{X}}_{j}$) and standard deviations (*S*_*i*_;*S*_*j*_); the confidence interval is calculated on a Microsoft Excel spreadsheet using the formula:

$$I_{ij}=\left[{\hat{X}}_{i}-{\hat{X}}_{j}-t_{2N-2;1-\alpha }\sqrt{\frac{S_{i}^{2}+S_{j}^{2}}{N}};{\hat{X}}_{i}-{\hat{X}}_{j}+t_{2N-2;1-\alpha }\sqrt{\frac{S_{i}^{2}+S_{j}^{2}}{N}}\right]$$

*t *_2*N*-2;1-*α*_:1-*α *quantile of the two-tails Student t-distribution with 2N-2 degrees of freedom.

## List of abbreviations

NF, Normalizing Factor; Ct, Cycle Treshold; PCR, Polymerase Chain Reaction; qPCR, quantitative PCR; sqPCR, semi-quantitative PCR; RT, Reverse Transcription; GAPDH, glyceraldehyde-3P-dehydrogenase; CCI, Controlled Cortical Injury; CHI, Closed Head Injury; FPI, Fluid Percussion Injury.

## Authors' contributions

HR performed all the in vitro experimental procedures and the data analysis, and was primary author of the manuscript. VE and DS supervised the study design and contributed to writing the manuscript. MP and C M-L provided the experimental model of traumatic brain injury. NC performed the experimental trauma.
